# Mechanisms of endothelial activation, hypercoagulation and thrombosis in COVID-19: a link with diabetes mellitus

**DOI:** 10.1186/s12933-023-02097-8

**Published:** 2024-02-20

**Authors:** Inés Valencia, Jairo Lumpuy-Castillo, Giselle Magalhaes, Carlos F. Sánchez-Ferrer, Óscar Lorenzo, Concepción Peiró

**Affiliations:** 1 Molecular Neuroinflammation and Neuronal Plasticity Research Laboratory, Hospital Universitario Santa Cristina, IIS Hospital Universitario de La Princesa, 28009 Madrid, Spain; 2 Laboratory of Diabetes and Vascular Pathology, IIS-Fundación Jiménez Díaz, 28040 Madrid, Spain; 3 Spanish Biomedical Research Centre On Diabetes and Associated Metabolic Disorders (CIBERDEM) Network, Madrid, Spain; 4 Department of Pharmacology, School of Medicine, Universidad Autónoma de Madrid, 28029 Madrid, Spain; 5 Vascular Pharmacology and Metabolism (FARMAVASM), IdiPAZ, Madrid, Spain

**Keywords:** COVID-19, SARS-CoV-2, Endothelial cells, Coagulation, Thrombosis, Diabetes mellitus

## Abstract

Early since the onset of the COVID-19 pandemic, the medical and scientific community were aware of extra respiratory actions of SARS-CoV-2 infection. Endothelitis, hypercoagulation, and hypofibrinolysis were identified in COVID-19 patients as subsequent responses of endothelial dysfunction. Activation of the endothelial barrier may increase the severity of the disease and contribute to long-COVID syndrome and post-COVID sequelae. Besides, it may cause alterations in primary, secondary, and tertiary hemostasis. Importantly, these responses have been highly decisive in the evolution of infected patients also diagnosed with diabetes mellitus (DM), who showed previous endothelial dysfunction. In this review, we provide an overview of the potential triggers of endothelial activation related to COVID-19 and COVID-19 under diabetic milieu. Several mechanisms are induced by both the viral particle itself and by the subsequent immune-defensive response (i.e., NF-κB/NLRP3 inflammasome pathway, vasoactive peptides, cytokine storm, NETosis, activation of the complement system). Alterations in coagulation mediators such as factor VIII, fibrin, tissue factor, the von Willebrand factor: ADAMST-13 ratio, and the kallikrein-kinin or plasminogen-plasmin systems have been reported. Moreover, an imbalance of thrombotic and thrombolytic (tPA, PAI-I, fibrinogen) factors favors hypercoagulation and hypofibrinolysis. In the context of DM, these mechanisms can be exacerbated leading to higher loss of hemostasis. However, a series of therapeutic strategies targeting the activated endothelium such as specific antibodies or inhibitors against thrombin, key cytokines, factor X, complement system, the kallikrein-kinin system or NETosis, might represent new opportunities to address this hypercoagulable state present in COVID-19 and DM. Antidiabetics may also ameliorate endothelial dysfunction, inflammation, and platelet aggregation. By improving the microvascular pathology in COVID-19 and post-COVID subjects, the associated comorbidities and the risk of mortality could be reduced.

## COVID-19 and the disruption of hemostasis

**Fig. 1:**
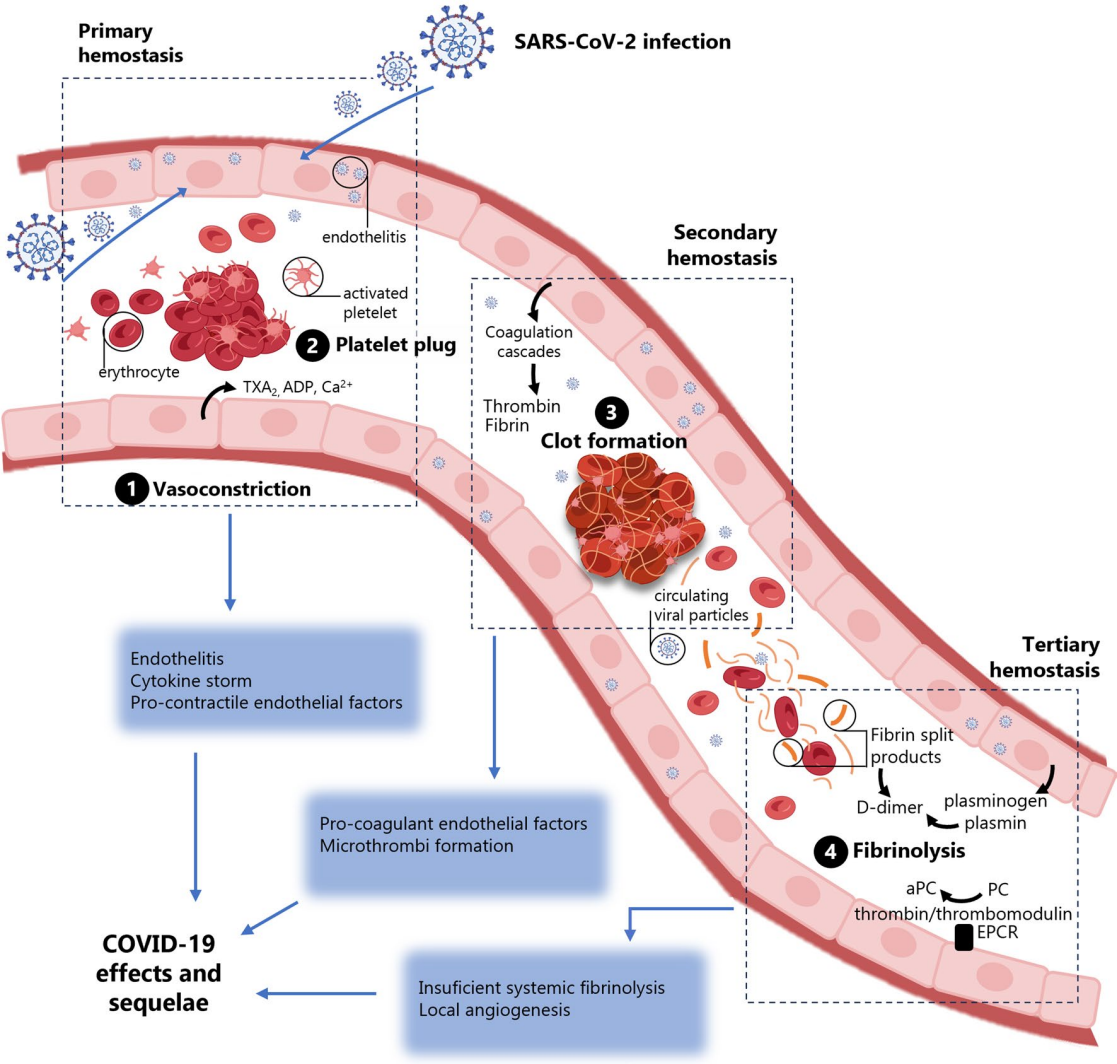
**COVID-19 and hemostasis regulation**. SARS-CoV-2 may infect endothelial cells (EC) causing endothelitis and directly disrupting endothelial homeostasis, leading to cytokine release, and favoring a pro-coagulant micro-environment. Then, primary hemostasis can be induced by fast vasoconstriction and release of pro-inflammatory and pro-contractile endothelial factors. Activation of coagulation cascades weaves thrombin and fibrin networks that immobilize erythrocytes and activated platelets to from a blood clot in the secondary hemostasis. The resolution of coagulation (tertiary hemostasis) may be also damaged in COVID-19 by alterations in the plasminogen-plasmin and thrombin/thrombomodulin-EPCRP-aPC pathways. Hyperinflammation, hypercoagulation, and hypofibrinolysis could be responsible for thrombotic events in COVID-19 subjects. TXA_2_ (thromboxane A2), aPC (activated protein C), PC (protein C), EPCR (endothelial protein C receptor)

Hemostasis is a finely tuned physiological process that leads to the cessation of bleeding from a blood vessel. It begins with trauma to the lining of the vessel wall and involves multiple interlinked steps that allow formation of a fibrin clot, which finally dissolves after injury is repaired. However, alterations in these processes may lead to hemostasis diseases [1, 2]. In this sense, COVID-19 infected patients have exhibited higher risk of arterial or venous thrombosis associated with disease severity [3] (Fig. 1). Higher plasma levels of hemostatic markers were linked to a worse prognosis and higher mortality [4]. Also, 25–85% of COVID-19 patients admitted to the intensive care unit (ICU) experienced a thrombotic complication, and postmortem analysis revealed the presence of endothelitis [5–7]. Importantly, both hypercoagulation and thrombotic events have been observed not only in acute COVID-19. They have been experienced in some few patients suffering from long-COVID syndrome [8], what is currently acknowledged as a challenging component of post-COVID-19 sequelae [9, 10]. Moreover, thrombocytopenia or thrombocytopenic purpura have been rare but serious adverse effects of SARS-CoV-2 spike (S) protein mRNA-based vaccines [11, 12] (see later).

Interestingly, among different regulators, the endothelium and its highly specialized EC placed at the inner layer of blood vessels tightly regulate vascular reactivity, cell growth, and inflammation by releasing and modulating specific factors [13]. Endothelial activation and dysfunction can be crucial for COVID-19 patients and its major related comorbidities like Diabetes Mellitus (DM) [14–16]. This narrative review aims to discuss the main triggers of endothelial dysfunction in COVID-19 and how subsequent disruption in endothelium-dependent hemostasis may promote hypercoagulation and thrombosis. This endothelial dysfunction can be a common event linking the hemostatic alterations observed in COVID-19 with those present in DM. Novel opportunities for therapeutical interventions will be also explored.

## Endothelial cell dysfunction and hypercoagulation in COVID-19

Besides pulmonary complications, endothelial injury was established as a primary finding in patients infected by SARS-CoV-2. This virus leads to a complex and multifactorial EC activation, progressive loss of anti-thrombotic factors, and promotion of local pro-angiogenesis (Fig. 1). Postmortem histology revealed viral inclusions in apoptotic EC, infiltration of inflammatory immune cells around the vessels and endothelial layer, and microvascular lymphocytic endothelitis [17, 18]. Clinical observations have identified the vasculature as one of the main trans-organ systems affected by SARS-CoV-2 infection as well as a major trigger of sequelae following COVID-19 [19]. In fact, viral tropism for vascular lesions has been identified in the most severe cases of COVID-19 [18]. While the endothelium basally favors an anti-thrombotic environment by preventing platelet activation and the onset of the coagulation cascade, both physiological and pathological stimuli may shift this balance towards pro-thrombotic and hypercoagulative states [20]. Indeed, presence of SARS-CoV-2 particles undergoes EC activation. A large body of clinical and experimental evidence currently supports the crucial role for EC activation in the pathological changes induced by SARS-CoV-2 in different territories, particularly, in terms of inflammation and thrombotic alterations [21–23]. As the intensity and/or the duration of the activation increases, the endothelial dysfunction, as an early sign of vascular disease, takes place.

Endothelial dysfunction runs with vasoconstriction, hyperpermeability, loss of integrity of the endothelial layer, and over-production of chemokines and cytokines together with upregulation of adhesion molecules for leukocytes [20, 24]. Activation of the endothelial monolayer also implies a phenotypic change from an anti-thrombotic to a pro-thrombotic surface more prone to platelet adhesion, together with deregulated synthesis and release of hemostatic factors and the onset of fibrin clots [20, 24]. Enhanced levels of adhesion molecules as well as platelet hyperactivation were observed in COVID-19 in correlation with severity of disease [25]. Thus, COVID-19-related vascular complications, including lung injury, stroke, myocardial dysfunction, or deep vein thrombosis, among others, share the common basis of endothelial dysfunction [20].

## Endothelial cell activators in COVID-19

**Fig. 2:**
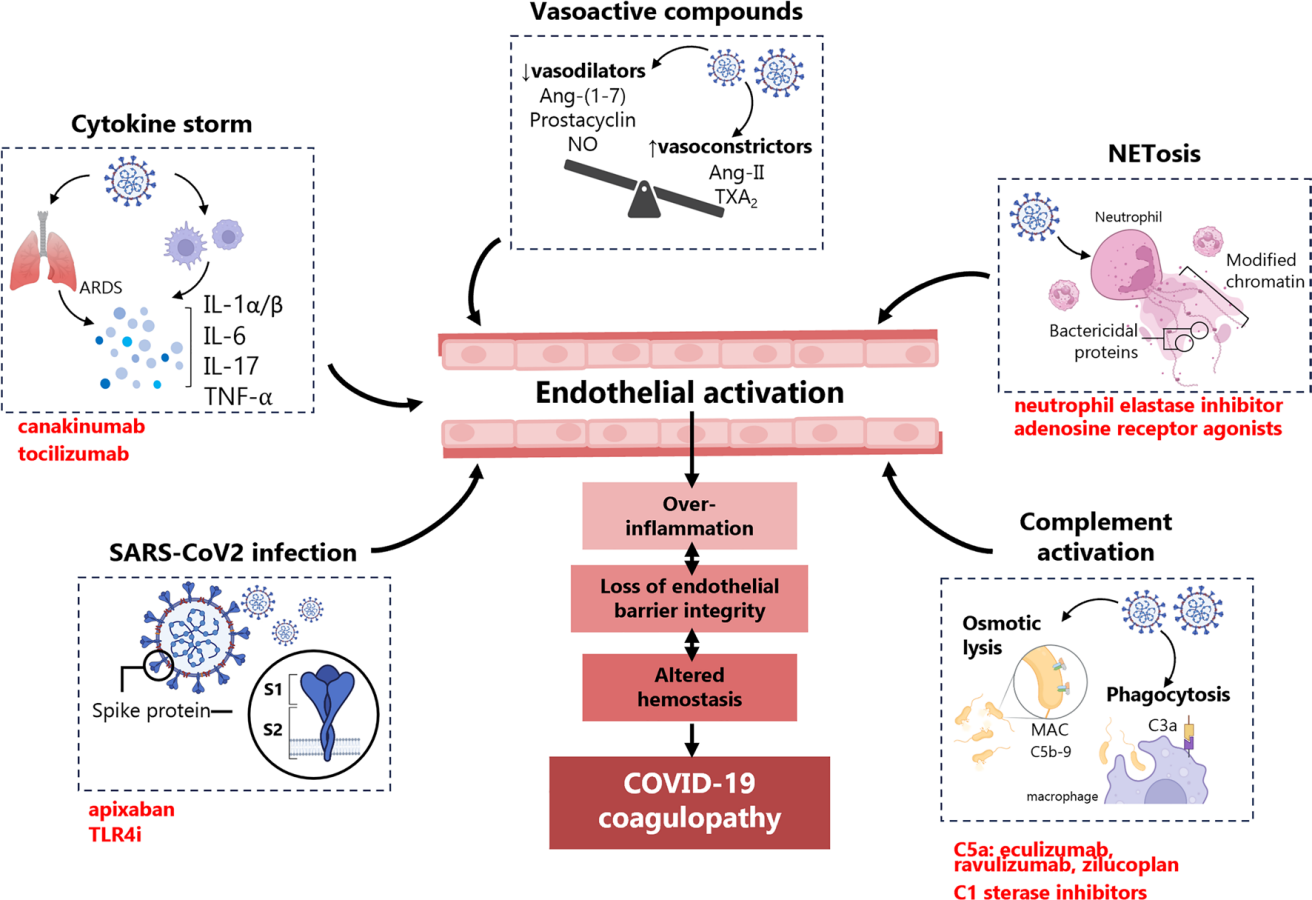
**The EC activation and dysfunction, as a central pathophysiological mechanism of COVID-19 coagulopathy**. SARS-CoV-2 infection and its concomitant local and systemic immunogenic stimuli (cytokine storm, vasoactive compounds, NETosis, and activated complement system) disrupt endothelial homeostasis leading to EC activation. This activation comprises over-inflammation, loss of endothelial barrier integrity and altered hemostasis, favoring coagulation and thrombosis. In red, specific drugs against mediators of endothelial activation. ARDS (acute respiratory distress syndrome), IL (interleukin), TNF-α (tumor necrosis factor alpha), Ang-(1-7) (angiotensin-(1-7)), NO (nitric oxide), Ang II (angiotensin II), TXA_2_ (thromboxane A2), MAC (membrane attack complex), TLR-4 (toll-like receptor 4)

The pathophysiological activation of EC in the context of COVID-19 is triggered by a variety of stimuli, including pro-inflammatory cytokines, vasoactive compounds, components of the immune system, or even by direct actions of SARS-CoV-2 and its isolated viral components (Fig. 2).

### Pro-inflammatory cytokines and vasoactive compounds

Endothelial activation becomes particularly relevant in acute COVID-19, where the acute respiratory distress syndrome (ARDS) and other complications are triggered by a cytokine storm, i.e., a burst of pro-inflammatory cytokines such as interleukin (IL)-6, IL-1α, IL-1β, IL-17, and tumor necrosis factor-alpha (TNF-α), among others [9, 26]. A large amount of clinical evidence shows a strong association between the inflammatory cell infiltration with increased thrombo-inflammatory biomarkers and COVID-19 severity [27, 28]. Enhanced levels of these cytokines, C-reactive protein, and ferritin were also associated with hyper-coagulation [29]. Moreover, the failure to resolve this inflammatory response could generate a cycle of unregulated events that contribute to endothelial activation and coagulopathy in acute and perhaps long-COVID [30]. In this regard, pro-inflammatory molecules such as platelet factor 4, α-2 antiplasmin, and the von Willebrand factor (WVF) were found increased in long-COVID, which may contribute to the failed fibrinolysis response and explain why these individuals suffer from constant fatigue, cognitive impairment, depression/anxiety, or dyspnea [31].

In addition, enhanced levels of vasoactive compounds, such as angiotensin II (Ang II) or thromboxane A_2_ (TXA_2_), which are potent vasoconstrictors and effectors of endothelial dysfunction with pro-inflammatory, pro-adhesive and pro-coagulant properties can play a role in the endotheliopathy associated to COVID-19 [32, 33] (Fig. 2). On the contrary, reduced levels or bioavailability of their physiological counterparts, such as angiotensin-(1–7), which is formed by a main receptor for SARS-CoV-2 (i.e., angiotensin converting enzyme 2; ACE2), prostacyclin, and nitric oxide (NO), can also contribute to the endothelial dysfunction and the hypercoagulation state [32, 34, 35].

### NETosis

An abnormal interrelation with immune components can also promote endothelial dysfunction. Neutrophils, as the largest population of myeloid leukocytes, are abundantly recruited in COVID-19 [36]. These phagocytes can act as endothelial activators through the release of extracellular neutrophil traps (NETs). NETs are generated by oxidative stress after stimulation of NADPH oxidase by NLRP3 inflammasome and pro-inflammatory cytokines (i.e., IL-1β and IL-18). Once released, NET components that include chromatin associated with bactericidal proteins from granules and cytoplasm, further intensify the pro-inflammatory response [37–41]. Although NETs mainly exhibit an antibacterial function, in excess, they can cause cell inflammation and tissue damage, increasing thrombogenicity of the endothelial layer [42, 43]. Indeed, NETs play an active role in the pathogenesis of coagulation and thrombosis of various origins by eliciting both extrinsic and intrinsic coagulation pathways [44]. In COVID-19, stimulated NET formation was associated with ARDS and hypercoagulability, as predictors of disease severity [42, 45] (Fig. 2).

### The complement system

The complement system, as an integral part of the innate immune response, also participates in the activation of the endothelium and contributes to the formation of a positive feedback loop between inflammation and thrombosis. The main activity of this system is to build up a multiprotein membrane attack complex (MAC) that ends with the death of pathogens by osmotic lysis or macrophage-mediated phagocytosis [46] (Fig. 2). In particular, high amounts of circulating C5a and soluble MAC (C5b-9), as well as processed fragments of C3, were seen in patients with severe COVID-19 [47–49]. The SARS-CoV-2 envelope proteins stimulate the lectin pathway of complement activation leading to C3 activation [50]. This activated C3a modulates the expression of endothelial adhesion molecules contributing to immune cell infiltration [51], and C5a directly stimulates secretion of IL-6, IL-8 and vascular endothelial growth factor (VEGF) [52]. Interestingly, a correlation between the activation of the complement and that of the endothelium has been also observed in chronic heart failure, suggesting the interrelated implication of both systems in vascular disease. In fact, complement and EC activation draw a bidirectional loop, since activated EC might also secrete complement components, mostly C7, and contribute to its plasmatic pool [53].

### SARS-CoV-2 and isolated viral components

Not only the pathophysiological responses triggered by SARS-CoV-2 have been identified as potential direct endothelial activators capable of triggering hemostatic abnormalities [54, 55]. Viral components like the S protein, formed by two domains named S1 and S2, are able to exert direct effects on EC by binding to cell surface receptors [56] (Fig. 2). Previous reports have described particles of SARS-CoV-2 inside EC [55, 57], and S1 presence was linked to endothelial dysfunction [56, 58]. We have recently observed a direct activation of endothelial pro-inflammatory pathways, including NF-κB and the NLRP3 inflammasome, and a disbalanced production of endothelial hemostatic regulators by the S protein [59]. Moreover, a persistent endothelial injury and inflammation were proposed as potential mechanisms of long-COVID and post-COVID sequelae related to cardiovascular events and dysregulated coagulation [60]. Indeed, in the arterial wall of severe COVID-19 patients, mRNA from SARS-CoV-2 was detected within endothelium, vascular smooth muscle, and infiltrated macrophages, representing a potential viral reservoir [18].

## Disbalanced endothelial-derived hemostatic regulators in COVID-19

After COVID-19, dysfunctional EC can synthesize and release several factors that are crucial regulators of coagulation and thrombosis [61]. The role of such regulators and how their delicate balance is disrupted in the context of COVID-19 is briefly reviewed over the next sections.

### The tissue factor

**Fig. 3:**
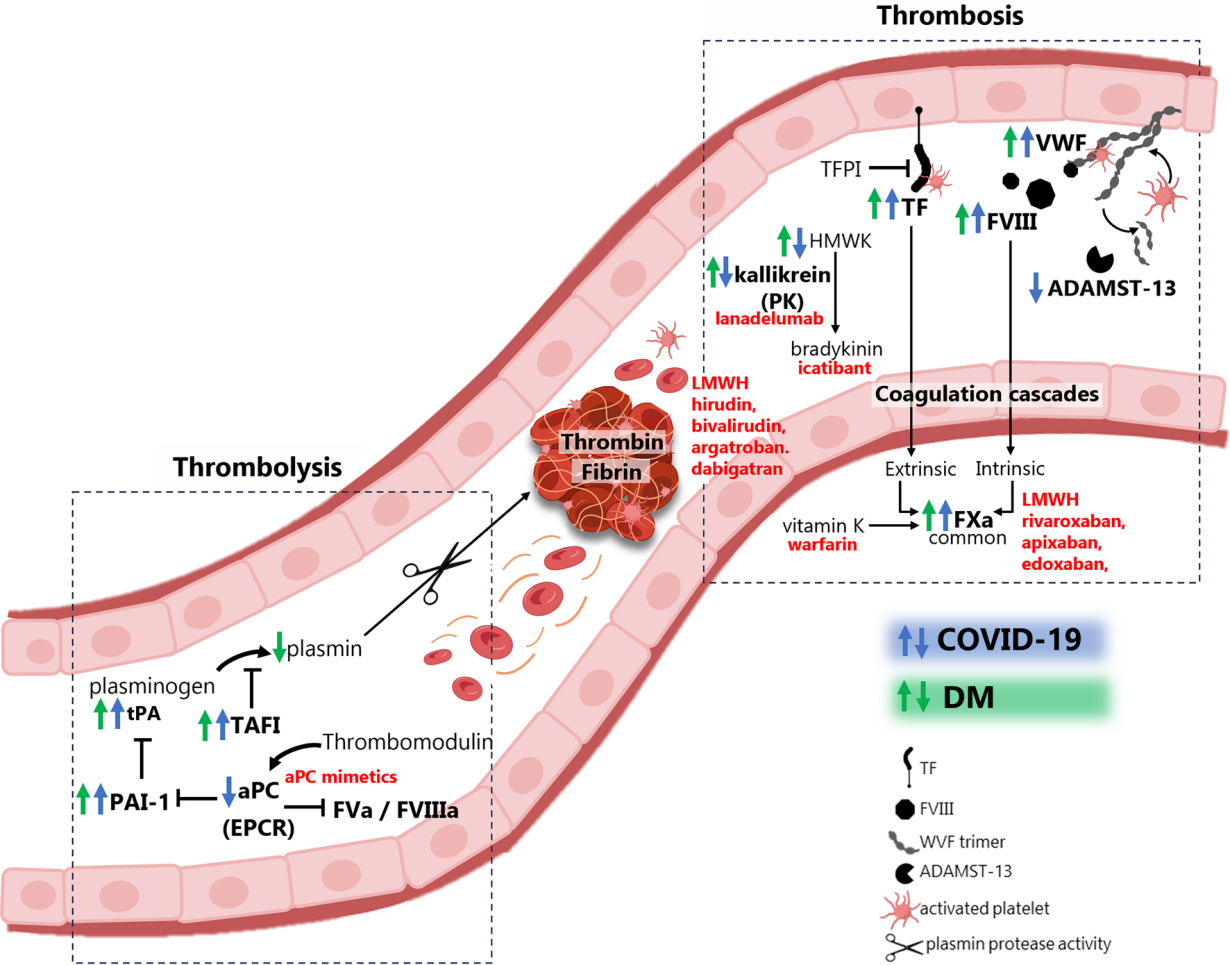
**Hypercoagulability and hypofibrinolysis in COVID-19 and DM**. Disrupted hemostasis during COVID-19 may be further intensified under diabetic milieu, resulting in a hypercoagulant phenotype of the activated EC. Elevated levels of pro-coagulant factors (VWF, FVIII, TF/TFPI, thrombin and fibrin) and diminished or insufficient anti-coagulant mediators (ADAMST-13, tPA-plasminogen, KKS, aPC-EPCR) may alter the thrombosis and thrombolysis equilibrium towards formation of blood clots. Arrows indicate over- or down-regulation of factors in COVID-19 (blue) or in DM (green) pathology. Drugs against specific mediators are shown in red beside its target of action. VWF (von Willebrand factor), FVIII (factor VIII), TF (tissue factor), HMWK (high molecular weight kininogen), PK (plasma kallikrein), LMWH (low molecular weight heparin), FXa (activated factor X), aPC (activated protein C), EPCR (endothelial cell protein C receptor), tPA (tissue plasminogen activator), PAI-1 (tissue plasminogen activator), TAFI (thrombin activatable fibrinolysis inhibitor)

The contact and intrinsic pathways may contribute to the pro-thrombotic state of COVID-19 [62]. The tissue factor (TF) is a transmembrane receptor that initiates the extrinsic coagulation cascade [63]. EC together with platelets, T lymphocytes, polymorphonuclear cells, monocytes, macrophages, dendritic cells, and fibroblasts are the main sources of TF [64–68]. Under physiological conditions TF is not typically expressed in active form by EC [69]. However, vessel injury and tissue trauma are major physiological activators of TF by promoting a change from an encrypted inactive to a decrypted active conformation. Then, TF acts as a high affinity receptor and cofactor for factor VII (FVII) and factor VIIa (FVIIa) at the site of tissue damage [70], and the TF:FVIIa complex activates factor X (FX) and factor IX (FIX), producing thrombin and fibrin, activation of platelet, and thrombosis [63]. To prevent excessive coagulation, EC also express tissue factor pathway inhibitor (TFPI) [71]. However, increased TF expression and fibrin enriched thrombi were reported in lung tissues from COVID-19 autopsies [72, 73] (Fig. 3). In human lung epithelial cells and EC, SARS-CoV-2 infection also enhanced TF expression and triggered pro-coagulant and pro-inflammatory responses [74, 75]. These actions were linked to the activation of the complement system, which could amplify and perpetuate endothelial dysfunction [41]. Also, the S protein inhibited TFPI and induced thrombogenic factors in human EC and neutrophils [55]. As consequence, higher circulating TF levels were associated with COVID-19 severity and associated mortality [76].

### Factor VIII

By non-covalent interaction in the Weibel-Palade (WP) bodies, factor VIII (FVIII) is protected from degradation by VWF [77, 78]. Once FVIII is activated to FVIIIa, it is detached from VWF and proteolized [79]. FVIIIa acts as a cofactor in the tenase complex (FIXa/FVIIIa), favoring conversion of FX to FXa [80]. Remarkedly, FVIII has been found disrupted in COVID-19 patients [81] (Fig. 3). Its plasma levels and activity were greatly elevated in parallel to disease severity [3, 81, 82]. In fact, FVIII was reported as an independent predictor of COVID-19 associated mortality [83].

Interstingly, the elevation of FVIII has been also reported in long-COVID in association with thrombo-inflammatory manifestations and vascular dysfunction [84, 85]. Also, very few cases of acquired hemophilia A, a bleeding condition caused by the development of auto-antibodies against FVIII, has been observed after SARS-CoV-2 S mRNA-based vaccination, perhaps as the result of a cross-reaction between antibodies against S protein and endogenous FVIII [86]. More commonly, antiphospholipid antibodies (aPL) derived from B1-cells induced up-regulation of C-reactive protein and D-dimer, and were related to fatal outcomes in COVID-19 patients. This lipid-binding aPL isolated from COVID-19 patients could target monocytes and EC to induce pro-thrombotic and inflammatory responses [87].

### The kallikrein-kinin system

In COVID-19, the intrinsic pathway of coagulation not only induces fibrin generation but also links to inflammation by release of kallikrein and bradykinin [88]. The kallikrein-kinin system (KKS) is a family of proteins that can effectively counteract blood pressure and eliminate pathogens by recruiting neutrophils to the site of injury. Kallikrein is mainly synthesized by the liver and endothelium (requiring factor XIIa; FXIIa) and released as plasma kallikrein (PK), which can be activated on the EC surface [89, 90]. Once active, kallikreins cleave kininogens (i.e., high molecular weight kininogen; HMWK) to ultimately synthesize bradykinin (BK) to mediates the release of NO and pro-inflammatory cytokines [91]. However, in COVID-19, the higher concentrations of some components of the KKS correlated with the severity of the disease [92] (Fig. 3). Thus, the hyperactivity of the KKS has become a prognostic marker of poorer outcomes in critically ill patients [93].

### The VWF: ADAMST-13 ratio

The VWF is a large multimeric glycoprotein synthesized and stored in EC and megakaryocytes/platelets [94]. After activation of EC or platelet, plasma VWF facilitate platelet aggregation and adhesion to the sites of vascular injury reinforcing the pro-coagulation effect of primary and secondary hemostasis [95]. The VWF form pro-coagulant multimers of different length interacting with angiopoietin-2 (Angpt-2) and osteoprotegerin at the WP bodies [96]. Remarkably, the VWF has been observed elevated in COVID-19 patients, acting as a marker of acute and sustained EC activation and predictor of poor outcomes [97, 98] (Fig. 3). Moreover, the VWF could be involved in local angiogenesis in severe COVID-19. Incubation of plasma from acute COVID-19 patients with EC triggered VWF secretion and Angpt-2 expression, as well as EC tube formation and angiogenesis [99].

In addition, one of the main regulators of the VWF activity is the disintegrin and metalloproteinase ADAMST-13, which is generated by hepatic stellate cells, but also from EC and megakaryocytes/platelets. ADAMST-13 cleaves VWF multimers reducing their pro-adhesive and pro-coagulant activity [100]. However, a higher VWF:ADAMST-13 ratio together with endothelial injury, coagulopathy, and poor prognosis has been found in acute and long-COVID syndrome [84, 101–103] (Fig. 3). In this line, COVID-19 vaccination could have originated pro-thrombotic complications in individuals with extremely low ADAMST-13 [104]. A deficiency in ADAMST-13 might be unable to counteract the VWF over-activity found after SARS-CoV-2 infection, long-COVID syndrome and, very rarely, COVID-19 vaccination [105].

### Fibrinolytic regulators

The fibrinolytic activity is essential to dissolve the fibrin clot in tertiary hemostasis and it is mainly determined by the balance between the endothelial tissue plasminogen activator (tPA) and its inhibitor, the plasminogen activator inhibitor type 1 (PAI-1) [106]. By producing tPA, the liver-derived plasminogen (PLG) converts into plasmin that eventually breaks up fibrin (releasing D-dimer) to dissolve blood clots. However, under SARS-CoV-2 infection endothelial dysfunction may also imbalance fibrinolysis [107] (Fig. 3). A time-dependent variation of the plasmin-dependent fibrinolytic system has been observed in COVID-19 progression, with initial activation followed by suppression in patients with more severe cases [108]. The plasma concentration of PAI-1 and the tPA-PAI-1 complex were elevated in COVID-19 subjects in comparison to healthy controls [109, 110], and the tPA levels at hospital admission were associated with lower survival rates [111]. Also, increased levels of the endothelial tPA receptor, annexin A2, correlated with inflammatory markers (i.e., IL-1β, IL-6, and TNF-α) and COVID-19 magnitude [112].

Furthermore, evidence suggests the existence of a circulating anti-coagulant factor, activated protein C (aPC) , which is triggered by the thrombin-thrombomodulin complex when bound to the EC protein C receptor (EPCR). aPC can suppress thrombin formation by proteolytical degradation or inactivation of coagulation factor Va (FVa) and FVIIIa and increases fibrinolytic activity by neutralizing PAI-1. However, the thrombin-thrombomodulin-EPCR complex is dysfunctional under COVID-19, affecting the aPC synthesis [113, 114]. Also, an enhanced thrombin generation, decreased fibrinolytic activity, and elevated levels of PAI-1 were identified in patients with long-COVID [115]. Likely, a complex disruption of the balance between thrombogenesis and thrombolysis is not properly solved in some cases or is excessively perpetuated in other patients. In this sense, micro-clots composed by fibrin amyloid and hyperactivated platelets might block capillaries and inhibit O_2_ transport to tissues, leading to some of the symptoms of the long-COVID and related post-acute sequelae [116].

In addition, elevated fibrinogen and D-dimer levels were correlated with hypercoagulable states, inflammation, and unfavorable outcomes after COVID-19 [117]. The increased D-dimer paradoxically coincided with decreased fibrinolytic capacity [117, 118]. Possibly, the presence of elevated D-dimer, especially during the early stages of pulmonary disease, could indicate the efforts of the local fibrinolytic system to eliminate fibrin and the necrotic tissue from the affected pulmonary parenchyma [118]. Thus, hyperfibrinolysis may be adequate at tissue level but systemically insufficient, delaying the resolution of fibrin deposition. In this context, the regulatory role of PAI-1 may further contribute to explain this effect since PAI-1, tPA, and the thrombin activatable fibrinolysis inhibitor (TAFI) augmented in parallel with COVID-19 severity [110, 119]. Further investigation on the interaction of pro-coagulant and anti-fibrinolytic factors may reveal novel issues in COVID-19 pathogenesis [120].

## Diabetes mellitus, a comorbidity in COVID-19

DM is a non-infectious epidemic disease whose prevalence in adults has risen from 108 million in 1980 to 537 million in 2021, and may reach 783 million by 2045 [121]. In fact, DM was responsible for 6.7 million deaths in 2021, a similar number to the fatalities associated to COVID-19 so far [122]. In this sense, the presence of DM has been recognized as a significant risk factor for the rapid progression and poor prognosis of COVID-19, ranging from 5.7% in non-severe cases up to 58% in critical individuals [123, 124]. More unfavorable outcomes have been observed in COVID-19-infected patients with DM than in those without DM. COVID-19 with DM leads to a twofold risk of ICU hospitalization and a two–threefold risk of mortality compared to COVID-19 alone [125, 126]. In a recent meta-analysis, which included 2,987,938 subjects with COVID-19, 10.4% of non-hospitalized individuals were diagnosed with DM, while DM was present in 28.9% of subjects requiring hospitalization and experiencing severe infection. In the same study, 34.6% of deceased patients exhibited both pathologies [127], and in another meta-analysis, the risk of mortality after infection was 54% higher in DM patients compared to non-DM patients [128]. Finally, a relative increased risk of 7–342% for the development of post-acute sequelae of COVID-19 has been reported in DM subjects. Thus, a potential correlation between DM and the long-COVID syndrome may affect vulnerable patients [129].

### Hyperglycemia and COVID-19

In the diabetic milieu, hyperglycemia can elicit changes in the immune system, favoring production and release of pro-inflammatory cytokines. After SARS-CoV-2 infection, serum levels of IL-6, C-reactive protein, ferritin, and D-dimer were significantly higher in DM patients compared to non-DM, enforcing the cytokine storm and contributing to the rapid deterioration of patients [130, 131]. In fact, hyperglycemic individuals treated with insulin showed lower risk to develop severe COVID-19. A poor glycemic control promoted higher levels of inflammatory markers during COVID-19 [130], and an adequate glycemic range (3.9–10 mmol/L) was associated with a reduction of adverse COVID-19 outcomes, including death [132]. In this regard, anti-hyperglycemic drugs decreased by 41% the rate of incidence for long-COVID syndrome [133]. Also, the level of SARS-CoV-2 replication was higher in the presence of serum from patients with DM than that from non-diabetics [134]. Thus, individuals with DM and COVID-19, even without other comorbidities, exhibit an increased risk of severe complications, such as pneumonia, uncontrolled inflammatory responses, hypercoagulability, and elevated mortality associated to dysregulated glucose metabolism [131].

On the other hand, SARS-CoV-2 infection may induce a diabetogenic actions. 17% of severe COVID-19 cases exhibited pancreatic lesions that could affect glycemic metabolism and inflammation [135, 136]. Administration of the S protein to type 2 DM-ACE2 knockout mice intensified cerebrovascular complications and cognitive dysfunction through activation of the renin–angiotensin–aldosterone system (RAAS) and toll-like receptor (TLR) signaling [137]. Individuals recovering from COVID-19 faced an elevated risk and burden of DM, leading to an increased usage of anti-hyperglycemic agents [135, 136]. Among fourteen studies that documented new-onset DM after COVID-19, twelve of them found a significant association between both pathologies, suggesting an increased risk of DM of 11–276% [129]. However, DM does not increase the risk of SARS-CoV-2 infection [138].

### Endothelial cell activation, a link between COVID-19 and diabetes

The coexistence of DM and COVID-19 could favor deleterious additive or synergistic effects related to endothelial dysfunction and coagulopathy (Fig. 3). In DM, endothelial activation and dysfunction underlies the associated cardiovascular complications. 80% of the cardiovascular deaths observed in DM are attributed to thrombotic events [139]. These responses are favored by factors also present in COVID-19. Pro-inflammatory cytokines may influence the disbalanced release of vasoactive factors and provoke over-activation of the complement system that amplifies endothelial dysfunction [140–143]. Moreover, COVID-19 associated NETosis can be also part of the pathogenesis of DM and its complications [144, 145]. In the other way, several stimuli related to the metabolic dysregulation can exert deleterious actions on the COVID-19 affected endothelium. The excess of plasma glucose and free fatty acids reduce NO and activate the NLRP3 inflammasome and NF-κB pathways favoring endothelial permeability [146, 147]. Stimulation of coagulation factors in DM may accentuate the risk of thrombotic events. In this sense, the thrombin-thrombomodulin-EPCR complex has been often observed dysfunctional under both DM and COVID-19, affecting aPC synthesis [113, 114]. Therefore, COVID-19 and DM, by sharing or accumulating mechanisms of endothelial activation and dysfunction, may stimulate more severe vascular-driven complications than these entities alone.

### Hemostasis disruption in DM and COVID-19

Hypercoagulability and hypofibrinolysis can be common features of both DM and COVID-19 due to the overactivation of the endothelium and variations of hemostatic factors [110, 148]. However, there is still limited evidence regarding how all hemostatic mediators vary in the combined context of these pathologies (Table 1). Through intricate interplays between the pathophysiological mechanisms inherent to both conditions, concomitancy of DM and COVID-19 could exacerbate inflammatory responses. As a result, subsequent alterations in hemostasis and coagulation worsen the disease progression and outcomes, leading to DM as a major risk factor for hemostasis disease in both acute and long-COVID patients [141, 149]. In fact, patients with both pathologies exhibited higher hypercoagulability and thrombotic complications than those infected patients without DM [150].

**Table 1:** Pro-coagulation and fibrinolytic factors in DM and COVID-19

Pathway	Coagulation Factors	DM	Severe COVID-19	DM + Severe COVID-19	References
A Secondary hemostasis					
**Extrinsic**	**Tissue factor**	↑	↑	n.r	[171, 172]
	**FVII**	↑ ↔	↑↓	n.r	[154, 156, 173, 174 ]
**Intrinsic**	**HMWK**	↑	↓	n.r	[93, 157]
	**PK**	↑	↓	n.r	[93, 175]
	**FXII**	↑↓	↓ ↔	n.r	[156, 173, 176, 177]
	**FXI**	↑ ↔	↓ ↔	n.r	[156, 173, 177, 178]
	**FIX**	↑ ↔	↔	n.r	[154, 156, 173]
	**FVIII**	↑	↑ ↔	n.r	[3, 81, 154, 174, 179]
	**VWF**	↑	↑ ↔	n.r	[97, 98, 160, 173]
**Common**	**FX**	↑	↑ ↔	n.r	[154, 173, 174]
	**FV**	↑	↑↓	n.r	[154, 173, 174]
	**Calcium**	↑	↑	n.r	[180, 181]
	**Prothrombin**	↑	↑	n.r	[182, 183]
	**Fibrinogen**	↑	↑	↑↑	[141, 184, 185]
	**FXIII**	↑	↓ ↔	n.r	[173, 186, 187]

Fibrinolytic Parameters	DM	Severe COVID-19	DM + Severe COVID-19	References
B Tertiary hemostasis				
**tPA**	↑	↑	n.r	[111, 112, 188, 189]
**Plasminogen**	↔	↑↓ ↔	n.r	[111, 173, 190, 191]
**Plasmin**	↓	↑↓	n.r	[111, 164, 192–194]
**D-Dimer**	↑	↑	↑↑	[4, 169, 195, 196]
**TAFI**	↑	↑	n.r	[110, 168]
**PAI-1**	↑	↑	n.r	[109, 110, 197, 198]

Variations of coagulation factors [Tissue factor (factor III, thromboplastin), FVII, HMWK (High molecular weight kininogen, Fitzgerald factor), PK (plasma kallikrein), FXII, FXI, FIX, FVIII, VWF (von Willebrand factor), FX, FV, Calcium (factor IV), prothrombin (factor II), fibrinogen (factor I), FXIII, tPA (tissue plasminogen activator), TAFI (thrombin-activatable fibrinolysis inhibitor), and PAI-1 (plasminogen activator inhibitor)] of secondary (**A**) and tertiary (**B**) hemostasis are described in DM, severe COVID-19, and DM-severe COVID-19 conditions. Arrows indicate over- or down-regulation of factors. n.r., non-reported data

Specifically, in primary hemostasis, alterations in platelet activation and aggregation, as well as in platelet interaction with endothelium were exacerbated in DM compared to control patients [151]. Hyperglycemia induced overexpression of vascular cell adhesion molecule-1 (VCAM-1) and P-, E- and L-selectins [152, 153]. Also, increased FVIII and other factors of the coagulation cascade, as well as the KKS activity were reported in patients with DM [154–157] (Table 1). Other authors demonstrated an elevation of TF in both type 1 and type 2 DM [158], or even after glucose variability [159]. Plasma VWF levels were also augmented in diabetics as compared to healthy controls, while ADAMST-13 was diminished [160, 161]. On tertiary hemostasis, up-regulated D-dimer and reduction of plasmin activity were described in DM [162–164]. Also, these patients displayed higher FXIII-induced crosslinking of plasmin inhibitor into the fibrin networks, and up-regulation of PAI-1 and TAFI [165–168].

Importantly, when both DM and COVID-19 overlapped, pro-coagulant factors and deficient fibrinolytic mechanisms were exacerbated. The combination of pre-existing hypofibrinolysis in DM with alterations in severe COVID-19 resulted in significant reduction in the body’s capability to dissolve clots. In these patients, higher levels of C-reactive protein and D-dimer were associated with lethality, and the cutoff value for D-dimer as a predictor of mortality was 2.8 ug/mL [169, 170]. Nevertheless, potential synergisms or additional mechanism of action for pro-coagulant and hypofibrinolytic profiles may be activated in diabetic COVID-19 subjects.

## Novel opportunities for therapeutic interventions

For COVID-19, the non-replicating adenoviral vectors were promising carriers for viral antigenic material (i.e., S protein) to induce safe and effective immunity against the virus. However, in 2021 several countries suspended vaccinations due to occurrence of vaccine-induced immune thrombotic thrombocytopenia events [199]. Some vaccines favoured hypercoagulopathy in specific patients with anatomical variants of cerebral venous outflow by inducing a transient inflammatory response and endothelial activation [200]. Although this thromboembolic complication was very rare, more preventive and therapeutic approaches could be suggested, particularly in patients with pro-coagulant comorbidities such as DM [201].

Therapeutic approaches for COVID-19 may depend on disease evolution and symptoms, as well as presence of complications and comorbidities. Asymptomatic infection includes patients with positive virologic test for SARS-CoV-2 but who have no symptoms consistent with COVID-19 [202]. Mild illness comprises individuals with several symptoms such as fever, cough, loss of taste and smell, muscle pain, and diarrhea, but not dyspnea. Herein, only patients aged ≥ 50 years old or with underlying comorbidities are at higher risk of disease progression [203]. In this sense, moderate illness, which includes subjects with lower respiratory disease, requires anti-SARS-CoV-2 treatment (antiviral, immunomodulator, anti-coagulant) [202]. In severe illness, patients show oxygen saturation < 94%, a respiratory rate > 30 breaths/min, a ratio of arterial partial pressure of oxygen to fraction of inspired oxygen < 300 mmHg, or lung infiltrates > 50% [203] . These patients can rapidly exhibit clinical deterioration and require additional oxygen therapy [202]. In critical illness, individuals exhibit ARDS, virus-induced distributive (septic) shock, cardiac shock, an exaggerated inflammatory response, thrombotic disease, and exacerbation of underlying comorbidities such as DM. They are admitted at the ICU to receive treatment for COVID-19 and comorbidities. In addition, reinfection with SARS-CoV-2 may occur as initial immune responses to the primary infection wane over time [204]. Data regarding the prevalence, risk factors, timing, and severity of reinfection likely vary depending on the SARS-CoV-2 variants. Nevertheless, no evidence suggests that the treatment should be different [205].

### Novel approaches for diabetic COVID-19

By unveiling the mechanisms that underlie the hypercoagulation/hypofibrinolytic responses in acute or long-COVID, novel therapeutic interventions might be suggested particularly favorable in high-risk patients like diabetics.

### Antidiabetics

Administration of insulin improved outcomes in patients with COVID-19 by achieving glycemic goals [130], but this hormone might increase mortality and complications in patients with both COVID-19 and DM [206]. However, metformin has exhibited therapeutic attributes beyond glycemic control. It ameliorated endothelial dysfunction by reduction of ROS production, the activation of NLRP3 inflammasome pathway, and downregulation of pro-inflammatory and adhesion molecules [207–210]. Also, metformin showed anti-thrombotic actions by attenuation of TF and platelet activation [211, 212]. Thus, it emerges as a promising candidate for enhancing survival in diabetic COVID-19 subjects [213]. Indeed, a recent meta-analysis established a correlation between metformin usage and reduction of mortality among diabetic COVID-19 subjects [214]. Metformin can also limit the replication of SARS-CoV-2 and the inflammatory response mediated by its S1 protein [215, 216]. Interestingly, although it did not reduce mortality nor ICU admission rates in non-diabetic patients with COVID-19 [217], outpatient treatment demonstrated an absolute reduction of 4.1% in the incidence of long COVID-19 [133]. Thus, positive effects of metformin may be also linked to modulatory effects on immune and hemostatic responses [218]. Other antidiabetics such as dipeptidyl peptisase-4 (DPP-4) and sodium-glucose cotransporter 2 (SGLT2) inhibitors, glucagon-like peptide-1 (GLP-1) agonists, thiazolidinediones, and sulfonylureas also reduced the risk of mortality in diabetic patients with COVID-19 [219, 220]. However, some contradictory results have been also reported since administration of DPP-4 inhibitors and sulfonylureas was associated with adverse outcomes and mortality in COVID-19 patients [220, 221].

### Immune-thrombotics

Multiple-target strategies may effectively dampen the immune-thrombotic response. The currently established anti-coagulant therapies include low-molecular-weight heparin (LMWH), warfarin, thrombin inhibitors (i.e. hirudin, bivalirudin, argatroban, dabigatran), or FXa inhibitors (i.e. rivaroxaban, apixaban, edoxaban) [222, 223]. Apixaban, a direct oral anti-coagulant, not only blocked both the free and clot-bound FXa and the activity of prothrombinase, it also inhibited the activity of SARS-CoV-2 protease M implicated in viral replication [224].

Novel approaches may target the complement system. Monoclonal antibodies like eculizumab, ravulizumab or zilucoplan inhibited the C5 cleavage or block the resulting fragments reducing generation of the MAC [225], and PK  and FIXa [88]. Inhibitors of C1 esterase are currently being assayed [225], and inhibitors of KKS, such as lanadelumab attenuated both the cytokine storm and hypercoagulation by blocking the PK activity. Also, antagonists for the bradykinin B2 receptor (i.e., icatibant) might be use for acute COVID-19 and perhaps for long-term manifestations of the disease [226]. In these cases, a major therapeutical challenge may consist in clearing viral particles from tissues or at least interfering with their potential receptors and signaling pathways. The viral S protein has been found in post-COVID tissues as a direct pro-inflammatory and pro-coagulant trigger of EC by interacting with a number of cell surface receptors including TLR-4 [227], [228]. In this sense, several TLR-4 inhibitors may be tested in this population [229–231]. Moreover, administration of aPC attenuated organ dysfunction and host death caused by ischemia–reperfusion in brain, heart, kidney, and lung in COVID-19 and other pathologies [232]. Inhibitors of the neutrophil elastase, which is released to the extracellular medium upon NETs formation, or agonists for the adenosine, which produce cyclic AMP, may mitigate NETosis [233–235]. The use of anti-cytokine drugs (i.e., against ILs, type-1 interferon (IFN)-γ) reduced the cytokine storm in COVID-19 [236, 237], and could indirectly attenuate NETosis and the complement system. In this sense, tocilizumab was approved by FDA as a candidate treatment of severe hospitalized COVID-19 patients [238], and canakinumab lowered the use of antidiabetic drugs in patients with COVID-19 and DM inducing prolonged reduction of systemic inflammation [239].

## Limitations

Despite numerous efforts to understand the variability in the severity of the disease, the specific relationship between different variants of SARS-CoV-2 and their impact on diabetic patients is still not fully elucidated. More comprehensive and coordinated research is needed, including genomic analysis, clinical studies, and epidemiological investigations, to better understand the connection between SARS-CoV-2 variants and the severity of infection in individuals with DM [240]. In addition, other limitation of this review is the selective use of literature, unavoidable because of the huge number of papers that emerged during and after this pandemic.

## Conclusions

Thrombotic complications are leading causes of hospitalization and death among COVID-19 and post-COVID-affected patients. Those DM patients infected with SARS-CoV-2 may exhibit exacerbated alterations in primary, secondary, and tertiary hemostasis by induction of endothelitis and endothelial dysfunction. As consequence of virus infection and the immune-defense response, increased activation of NF-κB/NLRP3 inflammasome pathways, vasoactive peptides, cytokines, NETosis, and the complement system, finally damage endothelial vasculature and stimulate coagulation mediators. This hypercoagulable state is favored by the lack of fibrinolytic factors, affecting blood irrigation in all tissues. However, several anti-coagulant therapies might be beneficial for these patients, however, therapeutic approaches reducing the initial triggers of pathological endothelial activation (i.e., by antidiabetics, immune-thrombotics) may improve vascular function and ameliorate risk of COVID-19 associated comorbidities.

## Data Availability

Not applicable.

## Abbreviations

ACE2: Angiotensin converting enzyme 2
Angpt-2: Angiopoietin-2
Ang II: Angiotensin II
aPC: Activated protein C
ARDS: Acute respiratory distress syndrome
aPL: Antiphospholipid antibodies
BK: Bradykinin
DM: Diabetes mellitus
DPP-4: Dipeptidyl peptidase-4
EC: Endothelial cell
EPCR: Endothelial cell protein C receptor
FVIII: Factor FVIII
GLP-1: Glucagon like peptide-1
HMWK: High molecular weight kininogen
ICU: Intensive care unit
IL: Interleukin
IFN: Type-1 interferon
KKS: Kallikrein-kinin system
LMWH: Low molecular weight heparin
MAC: Membrane attack complex
MCP-1: Monocyte chemoattractant protein-1
NETs: Extracellular neutrophil traps
NO: Nitric oxide
PAI-1: Plasminogen activator inhibitor type 1
PK: Plasma kallikrein
PLG: Liver-derived plasminogen
RAAS: Renin–angiotensin–aldosterone system
ROS: Reactive oxygen species
TAFI: Thrombin-activatable fribrinolysis inhibitor
SGLT2: Sodium-glucose cotransporter-2
TF: Tissue factor
TFPI: Tissue factor pathway inhibitor
TNF-α: Tumor necrosis factor alpha
tPA: Tissue plasminogen
TXA_2_: Thromboxane A2
TLR-4: Toll-like receptor 4 - S (spike protein)
VCAM-1: Vascular cell adhesion molecule-1
VEGF: Vascular endothelial growth factor
VWF: Von Willebrand factor
WP: Weibel-Palade
